# COMPASS: deCOMPressing stomA and two-Stage elective resection vs. emergency reSection in patients with left-sided obstructive colon cancer

**DOI:** 10.1186/s13063-023-07636-y

**Published:** 2023-10-05

**Authors:** Mathieu Pecqueux, Marius Distler, Olga Radulova-Mauersberger, Ulrike Neckmann, Sandra Korn, Christian Praetorius, Johannes Fritzmann, Anna Klimova, Jürgen Weitz, Christoph Kahlert

**Affiliations:** 1 Department of Visceral, Thoracic and Vascular Surgery, Faculty of Medicine and University Hospital Carl Gustav Carus, Technische Universität Dresden, Fetscherstrasse 74, 01307 Dresden, Germany; 2 National Center for Tumor Diseases (NCT/UCC), Dresden, Germany: German Cancer Research Center (DKFZ), Heidelberg, Germany; Faculty of Medicine and University Hospital Carl Gustav Carus, Technische Universität Dresden, Dresden, Germany; Helmholtz-Zentrum Dresden-Rossendorf (HZDR), Dresden, Germany

**Keywords:** Colon cancer, Colorectal cancer, Obstruction, Emergency, Randomized controlled trial

## Abstract

**Background:**

Colorectal cancer stands as a prevalent cause of cancer-related mortality, necessitating effective treatment strategies. Acute colonic obstruction occurs in approximately 20% of patients and represents a surgical emergency with substantial morbidity and mortality. The optimal approach for managing left-sided colon cancer with acute colonic obstruction remains debatable, with no consensus on whether emergency resection or bridge-to-surgery, involving initial decompressing stoma and subsequent elective resection after recovery, should be employed. Current studies show a decrease in morbidity and short-term mortality for the bridge-to-surgery approach, yet it remains unclear if the long-term oncological outcome is equivalent to emergency resection.

**Methods:**

This prospective, randomized, multicenter trial aims to investigate the management of obstructive left-sided colon cancer in a comprehensive manner. The study will be conducted across 26 university hospitals and 40 academic hospitals in Germany. A total of 468 patients will be enrolled, providing a cohort of 420 evaluable patients, with an equal distribution of 210 patients in each treatment arm. Patients with left-sided colon cancer, defined as cancer between the left splenic flexure and > 12 cm ab ano and obstruction confirmed by X-ray or CT scan, are eligible. Randomization will be performed in a 1:1 ratio, assigning patients either to the oncological emergency resection group or the bridge-to-surgery group, wherein patients will undergo diverting stoma and subsequent elective oncological resection after recovery. The primary endpoint of this trial will be 120-day mortality, allowing for consideration of the time interval between diverting stoma and resection.

**Discussion:**

The findings derived from this trial possess the potential to reshape the current clinical approach of emergency resection for obstructive left-sided colon cancer by favoring the bridge-to-surgery practice, provided that a reduction in morbidity can be achieved without compromising the oncological long-term outcome.

**Trial registration:**

German Clinical Trials Register (DRKS) under the identifier DRKS00031827. Registered on May 15, 2023.

Protocol: 28.04.2023, protocol version 2.0F.

**Supplementary Information:**

The online version contains supplementary material available at 10.1186/s13063-023-07636-y.

## Introduction

Colorectal cancer is the third leading cause of cancer death in both men and women in the USA [1] and the second leading cause of cancer death in men and the third leading cause of cancer death in women in Europe [2]. Worldwide, colorectal cancer accounts for 19.0 million (18.5–19.5) disability-adjusted life years (DALYs) [3]. The risk of developing colorectal cancer increases with age. More than half of patients develop the disease after the age of 70; only about 10% of cancers occur before the age of 55. Approximately 20% of patients initially present with acute colonic obstruction, which represents one of the most common causes of surgical emergency [4, 5]. While the consensus for right-sided obstructive colon cancer is primary resection and ileocolic anastomosis [6], international guidelines do not agree on the optimal management of the much more common left-sided obstructive colon cancer [4]. The most recommended procedure for patients with left-sided obstructive colon cancer is the emergency resection (ER) with or without anastomosis, which is a risk factor for high morbidity and mortality [5, 7].

A study evaluating the Dutch surgical colorectal audit data between 2009 and 2013 found an overall mortality of 8.5% after emergency resection compared to 3.4% in elective surgery [5]. Furthermore, primary anastomosis was only achieved in 64% of emergency resections, with 5% end ileostomy and 21% end colostomy compared to over 90% primary anastomosis in elective surgery [8].

A major burden for patients after emergency colon cancer resection is the development of postoperative complications, prolonged intensive care unit stay, permanent stoma, and in-hospital death. Patients undergoing emergency resection for obstructive cancer have a significant risk of permanent stoma [8, 9] and up to 70% of stoma patients experience long-term dysfunction or complications that significantly reduce their quality of life [3, 10]. Finally, the socioeconomic burden of colon cancer must be considered, as the cost of care has increased significantly in recent years. Currently, 31% of costs are spent on complications, with a 196% increase in costs for patients with major complications [11]. Reducing mortality and morbidity after emergency resection for obstructive left-sided colon cancer could significantly reduce costs to the healthcare costs [12, 13].

Several studies have suggested two-staged approaches with primary bowel decompression, either through the use of self-expanding metallic stents (SEMS) or placement of a decompressing stoma (DS) as a bridge-to-surgery (BTS). This approach aims to relieve colonic obstruction and allows patients to undergo elective oncological hemicolectomy after recovery. The BTS approach is considered a feasible and safe alternative to emergency resection with the goal of reducing postoperative complications [7]. Previous studies have mainly focused on comparing SEMS versus emergency resection in obstructive left colon cancer. A recent representative meta-analysis by Jain et al. concludes that SEMS offers several advantages over emergency resection, including significantly lower rates of permanent stomas, decreased anastomotic leakage, and improved overall in-hospital mortality [7]. However, the placement of SEMS requires a high level of endoscopic expertise that may not be available in all hospitals. Moreover, there is still a lack of evidence regarding the oncological risks associated with microperforations during stenting. Several studies have suggested a potential increase in locoregional recurrence rates [14]. Conversely, the BTS approach, involving the placement of a decompressing stoma, presents a technically straightforward procedure that can be performed minimally invasively and universally in emergency scenarios [15].

To date, only one randomized study has compared the use of a decompressing stoma as a bridge to surgery with emergency resection. Despite the study’s limitations, including a small patient cohort (*n* = 121), an extended inclusion period (1978–1993), and the absence of minimally invasive techniques, Kronborg et al. observed a significant decrease in the incidence of permanent colostomies and a non-significant trend towards lower morbidity among patients undergoing a two-staged approach with a decompressing stoma [16].

A meta-analysis from 2015 summarizes the findings of eight comparative studies involving 2424 patients from 1977 to 2015 [17]. While the meta-analysis did not identify significant differences in 30-day mortality and morbidity between the treatment groups, patients who received a decompressing stoma before elective resection were more likely to undergo primary anastomoses and less likely to be left with a permanent stoma [17].

A recent retrospective propensity-score matched study by Veld, Tanis et al. demonstrated that the use of a decompressing stoma as part of the BTS approach resulted in a significantly decreased 90-day mortality rate compared to emergency resection (1.7% vs. 7.2%, *p* = 0.006) [18]. This difference was particularly pronounced in the patients over 70 years of age (3.5% vs. 13.7%, *p* = 0.027). Additionally, patients treated with a decompressing stoma underwent a higher proportion of minimally invasive resections (56.8% vs. 9.2%, *p* < 0.001) and had a greater incidence of primary anastomoses (88.5% vs. 40.7%, *p* < 0.001), a reduced occurrence of permanent stomas (23.4% vs. 42.4%, *p* < 0.001), and improved 3-year overall survival rates (79.4% vs. 73.3%, hazard ratio 0.36) [18].

These findings collectively suggest that the BTS approach for obstructive left-sided colon cancer has the potential to decrease perioperative mortality and improve quality of life by reducing the need for permanent stomas without compromising long-term oncological outcomes. However, it should be noted that the retrospective nature of the studies mentioned above precludes a comprehensive evaluation of dropout rates between decompressing stoma placement and subsequent resection. Thus, a prospective randomized trial is necessary to conduct an intention-to-treat analysis and validate these findings.

In this randomized multicenter trial, we aim to compare the outcomes of patients with obstructive left-sided colon cancer who undergo either emergency resection or the placement of a decompressing stoma as a bridge-to-surgery strategy.

## Methods

### Ethical approval and study registration

The COMPASS trial has obtained ethical approval from the ethical committee of the TU Dresden (Ethical Committee number EK-278062022). Furthermore, the trial has been registered on the 15 May 2023 in the German Clinical Trials Register (DRKS) under the identifier DRKS00031827 (https://drks.de/search/en/trial/DRKS00031827).

### Study design

**Fig. 1:**
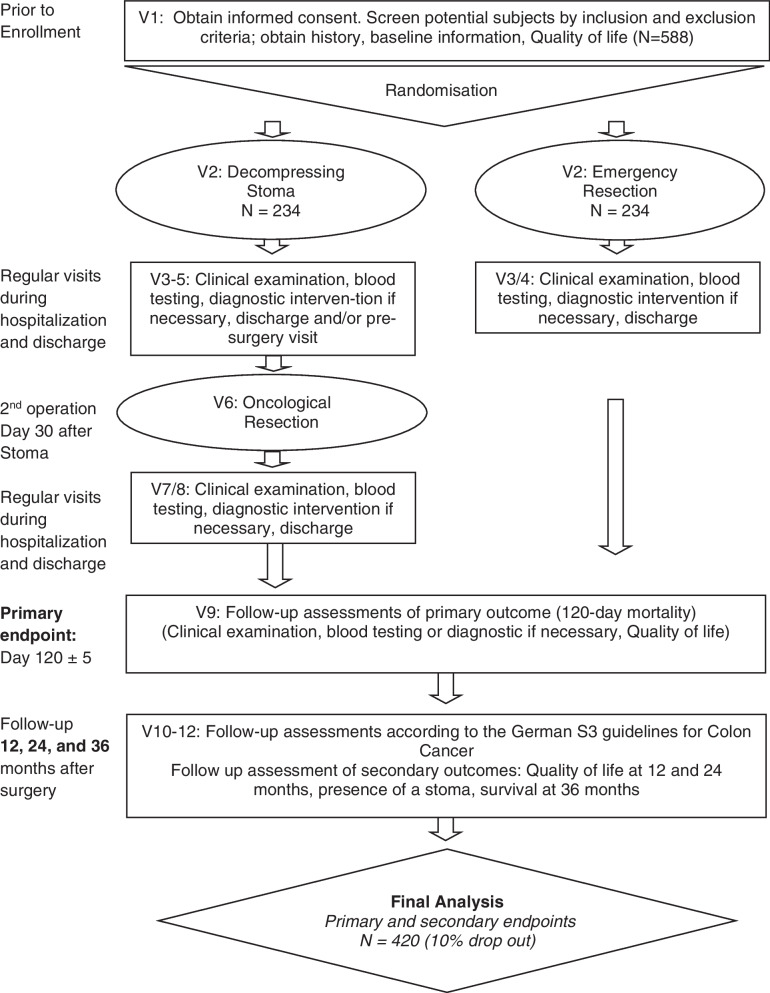
Flowchart of the trial scheme

The COMPASS trial is a multicenter, randomized, open-label study that will be conducted across 26 university hospitals and 40 academic hospitals in Germany. The trial was designed to show the superiority in regard to 120-day postoperative mortality among patients subjected to a two-stage bridge-to-surgery (BTS) procedure, as opposed to those undergoing a single-stage emergency resection (ER) for the management of obstructive left-sided colon cancer. Inclusion criteria involve patients presenting with symptoms of obstruction (abdominal distension, nausea, and/or vomiting) and having a left-sided colon tumor suspected of being colon cancer. Radiological assessments, including X-ray or a CT scan, must demonstrate a dilated colon with or without dilatation of the small intestine. Additionally, a high-grade suspicion of colon cancer should be identified on CT or endoscopy during the preoperative examination. Please refer to Fig. 1 for an illustration of the trial scheme.

### Stratification and randomization

Patient randomization will be performed using a secure online computer randomization system, either directly in the operating room or by the trial coordinator via phone. Block randomization will be employed to ensure balanced participant numbers throughout the trial. Stratification will be applied based on the following factors: age (younger or older than 70 years), tumor stage, gender, ASA classification (1–2 vs. 3), presence of small bowel distention, and study center. Randomization must be completed within 24 h of study enrollment. Treatment allocation will not be concealed from patients, physicians, or researchers at any point during the trial.

After obtaining preoperative informed consent, patients will be randomly assigned in a 1:1 ratio to the trial. The choice of the surgical technique (minimally invasive vs. open) and the method of reconstruction (primary anastomosis or discontinuous resection) during tumor resection, as well as the type of stoma (loop ileostomy, loop colostomy or cecal fistula) in the bridge-to-surgery group is at the discretion of the surgeon.

### Inclusion and exclusion criteria

Inclusion criteria:

- Patients with a left-sided colon or upper rectal tumor (from the splenic flexure to the intraperitoneal rectum with the tumor located more than 12 cm from the anal verge) undergoing curative treatment
- The tumor must be highly suspicious for colon cancer in CT or endoscopy
- Colonic dilatation confirmed by CT or X-ray
- The tumor, including potential metastases, must be deemed suitable for curative resection
  - ◦ Liver metastases must be deemed completely resectable (R0) during the primary tumor resection (subcapsular metastases or metastases requiring removal of no more than tree liver segments simultaneously with the primary tumor)
  - ◦ Pulmonary metastases that require maximum unilateral lobectomy for curative purposes without infiltration of the main bronchus or central pulmonary artery/vein
- Patients aged 18 years or older
- Written informed consent provided.
- No prior or concurrent malignancies within the 2 years preceding the enrollment

Exclusion criteria:

- Right-sided colon tumor
- Extraperitoneal rectal cancer of the lower and middle third (tumor located less than 12 cm from the anal verge)
- Life expectancy less than 120 days
- Locally advanced tumor disease with local infiltration of adjacent structures that precludes R0 resection or necessitates neoadjuvant treatment
- Patients receiving palliative treatment
- Evidence of bowel perforation on CT or X-ray
- Patients deemed ineligible for surgery (ASA score ≥ IV)
- Lack of compliance
- Presence of addiction or other medical conditions that hinder the individual’s ability to comprehend the nature and consequences of the clinical trial.

### Intervention

The aim of the current study is to compare two surgical approaches for the resection of left-sided obstructive colon cancer: the single-stage emergency resection (control group) and the two-stage bridge-to-surgery procedure (intervention group). Both groups will undergo curative hemicolectomy, including tumor resection and lymph node dissection. International guidelines provide varying recommendations, with 42% advocating emergency surgery and 47% suggesting either emergency surgery or stenting as bridge-to-surgery. Primary resection with or without anastomosis is the most commonly recommended procedure [5]. Accordingly, emergency resection is currently the most frequently performed approach in many countries [19]. Retrospective studies have indicated that the two-stage approach with decompressing stoma can reduce 90-day mortality from 7.2 to 1.7% and the incidence of permanent stomas from 42 to 23% compared to the control group [18]. Considering these factors, we have determined the one-stage approach with emergency resection of the tumor to be the control group, reflecting the prevailing practice. Conversely, the two-stage procedure with decompression of the colon before elective surgery, as recommended by some guidelines, is assigned as the intervention group.

While the BTS approach can improve the patient’s general condition during resection, it also prolongs the time-to-surgery (TTS). Prolonged TTS may have an impact on cancer survival. Studies by Satish et al. and Kaltenmeier et al. have presented conflicting results, with Satish et al. suggesting that extended TTS (> 21–30 days) is associated with a lower adjusted risk of death [20], while Kaltenmeier et al. found that prolonged TTS is associated with an increased risk of death [21].

To address this potential issue, our study design incorporates TTS as a secondary end point and evaluate its association with cancer-specific factors, such as cancer survival.

#### Control group (emergency resection arm)

In the control group, patients will undergo a single-stage oncological left-sided hemicolectomy with the option of a primary anastomosis with or without loop ileostomy or a Hartmann’s procedure (closure of the colorectal stump and end colostomy).

#### Intervention group (decompressing stoma as bridge-to-surgery arm)

In the intervention group, patients will undergo a two-stage resection with a decompressing stoma procedure. After a period of convalescence, an elective, oncologic left hemicolectomy will be performed. The stoma technique during the first procedure can be performed as either loop or blowhole colostomy (a loop ileostomy is also possible if deemed adequate), while the elective resection can be conducted as a primary anastomosis with or without loop ileostomy or a Hartmann’s procedure. The time interval between the decompressing stoma and resection will be determined by the treating surgeon, with an expected period of approximately 30 days based on current studies [18, 22].

Any additional concurrent care, including the use of adjuvant therapy after resection, is left to the discretion of the treating physicians.

### Endpoints of the study

The study has identified several primary and secondary outcomes to evaluate the effectiveness and impact of the two surgical approaches for left-sided obstructive colon cancer. The primary outcome focuses on perioperative mortality within a 120-day period after surgery. Usually, perioperative death is defined as mortality within 90 days after surgery, but to compensate for the delay between decompressing stoma placement and tumor resection in the intervention group, we opted to extend the primary endpoint by 30 days according to recent studies [14, 18, 22].

Primary outcome:

- Perioperative 120-day mortality
  - *Control group*: mortality within 120 days after emergency resection
  - *Intervention group*: mortality within 120 days after decompressing stoma placement

Secondary outcomes:

1. *Quality of life*
2. *Permanent Stoma rate*
3. *Stoma-associated complications*
4. *Quality-adjusted survival (QALY)*
5. *Number of resected lymph nodes*
6. *Number of R0 resections*
7. *3-year overall survival*
8. *3-year cancer-specific survival*
9. *3-year disease-free survival*

The secondary endpoints were developed in collaboration with experts as well as patient groups, such as the German ILCO, to ensure a comprehensive assessment of patient outcomes, including quality of life, complications, the rate of permanent stomas, and cancer-related factors.

### Statistics

The statistical analysis plan for the study involves various tests and methods to analyze different endpoints. The chi-squared test will be applied to compare the perioperative 120-day mortality between the control and intervention arms using an intent-to-treat analysis. The significance levels were set to 5% (two-sided). The secondary endpoints will each be analyzed accordingly: The rate of primary anastomoses, permanent stomas, and 120-day complication rate will be investigated using the chi-square test. The 3-year overall survival will be examined using the Kaplan-Meyer estimator combined with the log-rank test. The treatment effect on the patient-reported quality of life will be analyzed using a regression analysis. Quality-adjusted survival will be analyzed using marginal models and non-parametric regression.

The statistical significance of stratification factors will be assessed. Propensity score matching may be used as a part of a supplementary analysis, to control for potential confounding factors that were not accounted for during sampling. In particular, a propensity score matching procedure with age as a continuous variable may be used to control for between-arm age differences. Statistical analyses of matched-sub-cohorts will have an exploratory character, providing additional information about effect sizes. The standardized mean differences and confidence intervals will be reported, and *p*-values will be provided as an additional information.

### Sample size calculations

The sample size calculation was performed using an estimated 120-day mortality rates of 8% and 1.7% in the control and intervention arms, respectively. These rates were extrapolated from the 90-day mortality rates of 1.7% (decompressing stoma (DS)) vs 7.2% (emergency resection (ER)) as well as the 3-year overall survival data in the propensity-score matched study by Veld, Tanis et al. [18]. The effect size was then reduced with an assumed 120-day mortality rate of 1.7% for the decompressing stoma group and 7% in the emergency resection group, resulting in a reduced effect size of 0.274 and an increased relative risk of 0.243.

To achieve the 80% power with a (two-sided) 5% significance level using a normal curve via arcsine transformation (Cohen, 1988, Chapter 6), at least 210 patients per arm (total 420 patients) need to be analyzed. To account for an expected 10% dropout rate, the target enrollment is 468 patients (234 per arm). Considering a 20% screen-failure rate, 588 patients will need to be assessed for eligibility. The power calculations were performed using SAS Version 9.4.

### Methods against bias

To address potential bias and ensure the integrity of the study, several methods have been implemented. As mentioned above, randomization and stratification will be used to allocate patients into the control and intervention groups. This process will be conducted using a secure online computer randomization system and will consider factors such as age, tumor stage, gender, ASA classification, presence of small bowel distention, and study center. By stratifying these variables, a balanced distribution can be achieved, reducing the risk of bias. To account for the delay between decompressing stoma placement and tumor resection in the intervention group, the primary endpoint of perioperative mortality has been adjusted to 120-day. This extension allows for a comprehensive evaluation of mortality rates in both groups with an appropriate timeframe.

The primary endpoint will be analyzed using the intention-to-treat (ITT) principle, and the final study report, in line with transparent reporting standards, will adhere to the CONSORT (Consolidated Standards of Reporting Trials) statement.

### Feasibility of the recruitment

Based on the estimated incidence of acute large bowel obstruction in Germany, it is anticipated that approximately 2500–5000 patients per year would be eligible for the study [4, 5]. In our own Department of Surgery, approx. 15–20 patients/year present with a left-sided obstructive colon cancer. We estimate that our center could include around 10 patients per year into the study. Based on these data and the retrospective Dutch trial by Veld, Tanis et al. [18], we estimate that a minimum of 35 large centers are needed to achieve the required sample size. Currently, 66 hospitals have committed to participate in the trial. The participating centers were selected according to recruitment potential, existing trial infrastructure and medical experience. To ensure adequate representation across different levels of care, basic care hospitals, and hospitals of the regular and priority care as well as maximum care hospitals such as university hospitals will be included in the recruitment.

### Oversight and monitoring

The study’s regulatory and supervisory functions are managed by the principal investigator (PI) in conjunction with the Coordination Centre for Clinical Trials (KKS) Dresden. The KKS Dresden assumes the data monitoring, which will follow a risk-based approach in line with the ICH-GCP (R2) guidelines. This includes assessing feasibility, availability and completeness of study material, ethics committee approvals, and providing adequate training to local staff. Monitoring activities will also verify written consent forms, source data, and accurate data entries. The frequency of monitoring will be determined based on the assessed risk to maintain data quality and study integrity. A patient advisory council board, comprised of individuals affiliated with the patient self-help organization ILCO, will offer invaluable support to the principal investigator (PI) and the coordinating center by contributing their personal expertise and experience when confronted with challenges that emerge throughout the course of the clinical trial. By implementing these methods, the study aims to minimize bias, ensure rigorous data collection and analysis, and provide a comprehensive and reliable evaluation of the chosen surgical approaches.

An independent data and safety monitoring board (DSMB), comprised of members without conflict of interest with the project and study investigators, will conduct regular interim analyses to assess the progress of the study and examine safety variables. The DSMB can terminate the study for safety reasons or due to early superiority, without predefined definitions.

### Data management

Personal health data will be recorded (Table 1) and archived by authorized study personnel for a minimum of 10 years, ensuring privacy by removing identifying information. Pseudonymized data will be transmitted to the Clinical Study Center for analysis and monitoring, accessible only to authorized personnel bound by confidentiality agreements. Participants have the right to revoke their consent for data storage and use, with the option for pseudonymized data to be retained unless specifically requested for complete deletion. Data entry is performed using a study software that complies with the requirements of applicable laws and guidelines, particularly ICH-GCP. The extent of database access and associated permissions are regulated through corresponding user roles. Data completeness, plausibility, and consistency are checked through programmed validations directly in the electronic case report form (eCRF) as well as supplementary manual checks outside of the eCRF. Any queries arising from these checks are sent directly to the respective study center within the eCRF. Corrections resulting from the responses to these queries are made by the study center in the eCRF.

**Table 1:** Study assessment table

	**Baseline**	**Intervention**	**Follow-up**			
	**Visit 1/5**^a^	**Visit 2/6**^a^ (day of surgery)	**Visit 3/7**^**a**^ (POD 5 ± 1 days)	Visit 4/8* (day of discharge)	**Visit 9** (120 ± 7 days POD)	**Visits 10–12 (EOS)** (12, 24, and 36 months POD)
**Selection criteria informed consent**	•					
**Standardized imaging (radiography, CT)**	•				•	•
**Demographic data medical history ECOG status clinical-pathological features**	•					
**Laboratory tests**^b^	•		•			
**Serum CEA**	•				•	•
**Randomization**^c^	•					
**Study intervention**		•				
**Intraoperative results**		•				
**Postoperative results**			•	•	•	
**Quality of life**^d^	•				•	•
**Oncologic follow-up**					•	•

*CEA* carcinoembryonic antigen, *Ct* computed tomography, *EOS* end of study, *POD* postoperative day, *ECOG* Eastern Cooperative Oncology Group

^a^Intervention arm only

^b^Laboratory tests include: complete blood count, aspartate aminotransferase (AST; GOT), alanine aminotransferase (ALT; GPT), gamma-glutamyltransferase (γGT), lactate dehydrogenase (LDH), total bilirubin, creatinine, calculated glomerular filtration rate (GFR), albumin, and international normalized ratio (INR)

^c^Randomization must occur within 24 h after inclusion in the study at visit 1

^d^Quality of life will be assessed using the EORTC QLQ-C30 at visit 1/5* (baseline), visit 9 (POD 120), and visit 10–12 (12, 24, and 36 months after surgery)

### Criteria for discontinuing the study

The following termination rules or criteria are applicable:

1. Individual patient termination: In the event that a patient, who has previously undergone a decompressive stoma procedure within the intervention group, becomes medically ineligible for the subsequent two-stage operation, their informed consent will be withdrawn.
2. Exclusion of participating investigative centers: Investigative centers that deviate from the study protocol, primarily due to inadequate patient recruitment (less than one per year) and compromised data quality, will be excluded from the analysis.
3. Study suspension: The entire study will be temporarily suspended following the occurrence of any of the subsequent events until evaluated by the data and safety monitoring board (DSMB):
  Elevated incidence of severe morbidity and/or postoperative mortality observed within either the intervention or control group
  Increased frequency of surgical interventions within either the intervention or control group
  Receipt of a recommendation from the DSMB to prematurely terminate the study due to participant safety concerns in either of the two groups

### Protocol changes

Modifications to the study protocol will only be made upon obtaining prior approval from the ethical committee. Any protocol changes will be duly noted on the German Clinical Trials Register (DRKS).

### Dissemination trial results

The results will be submitted to the German Clinical Trials Register (DRKS) and published in a peer reviewed journal. Furthermore, the patient group ILCO which was involved in defining the primary and secondary endpoints of the study will be used to disseminate the results among its members.

## Discussion

Colorectal cancer represents the third leading cause of cancer death in Europe and the USA. The risk increases with age, with more than half of the patients diagnosed above the age of 70. Acute colonic obstruction is a common presentation, accounting for up to 30% of cases, and represents one of the most common causes of surgical emergency. While the preferred treatment for obstructing right-sided colon cancers is primary resection with an ileocolic anastomosis, there is a lack of consensus regarding the optimal approach for left sided obstructive colon cancer [4], which accounts for two thirds of all colon cancers [23]. Primary resection with or without anastomosis is the most recommended procedure according to international guidelines, although bridge-to-surgery treatment is also considered. Emergency resection carries a higher risk of morbidity and mortality, while the bridge-to-surgery approach has the potential for resection and intermediate metastasis.

A recent large retrospective study utilizing data of the Dutch Colorectal Audit (DCRA)—a national mandatory registry—suggests that the bridge-to-surgery approach can significantly reduce perioperative mortality and permanent stoma rates and improve long-term survival in patients with left-sided obstructive colon cancer [18]. However, retrospective studies have limitations in accurately representing the interval between stoma creation and resection. Furthermore, existing prospective studies are either very outdated [16] or involve a comparison between endoscopic stenting and emergency resection in a relatively small number of patients [24]. Endoscopic stenting in obstructive cancer is technically challenging with a limited success rate of 78% in highly specialized centers. This high level of endoscopic expertise is not available in most hospitals where patients with acute obstructive colon cancer present themselves. Additionally, concerns regarding microperforations during stenting and their impact on long-term oncological outcomes exist. Nevertheless, these studies have shown an increased rate of primary anastomoses and a significant reduction in permanent stomas.

To address these uncertainties, the present study aims to compare the peri- and postoperative outcomes of patients with obstructive left-sided colon cancer who undergo emergency resection versus those who receive decompressing stoma as bridge-to-surgery. The objective is to determine the optimal approach for the treatment of obstructive left-sided colon cancer and shed light on the associated perioperative and long-term outcomes.

## Trial status

COMPASS is currently establishing recruitment sites and is expecting to begin recruitment in October 2023. The current protocol version is 2.0F. The approximate end date for recruitment will be October 2026.

### Supplementary Information

**Additional file 1.** 

## Data Availability

The data that support the findings of this study are available from the corresponding author upon reasonable request.
