# Correction: COMPASS: deCOMPressing stomA and two-Stage elective resection vs. emergency reSection in patients with left-sided obstructive colon cancer

**DOI:** 10.1186/s13063-023-07722-1

**Published:** 2023-10-24

**Authors:** Mathieu Pecqueux, Marius Distler, Olga Radulova-Mauersberger, Ulrike Neckmann, Sandra Korn, Christian Praetorius, Johannes Fritzmann, Anna Klimova, Jürgen Weitz, Christoph Kahlert

**Affiliations:** 1 Department of Visceral, Thoracic and Vascular Surgery, Faculty of Medicine and University Hospital Carl Gustav Carus, Technische Universität Dresden, Fetscherstrasse 74, 01307 Dresden, Germany; 2 National Center for Tumor Diseases (NCT/UCC), Dresden, Germany

**Correction: BMC Trials 24, 641 (2023)**

**https://doi.org/10.1186/s13063-023-07636-y**

Following publication of the original article [1], we have been informed that the first and last names of the first two and last two authors have been switched.

Originally published: Pecqueux Mathieu, Distler Marius, Weitz Jürgen and Kahlert Christoph. Correct name tagging: Mathieu Pecqueux, Marius Distler, Jürgen Weitz, Christoph Kahlert

Also, there were originally five author affiliations declared for authors 1, 2, 3, 6, 7, 8, 9 and 10:

1 Department of Visceral, Thoracic and Vascular Surgery, University Hospital Carl Gustav Carus, Technische Universität Dresden, Fetscherstrasse 74, Dresden 01307, Germany. 2 National Center for Tumor Diseases (NCT/UCC), Dresden, Germany. 3 German Cancer Research Center (DKFZ), Heidelberg, Germany. 4 Faculty of Medicine and University Hospital Carl Gustav Carus, Technische Universität Dresden, Dresden, Germany. 5 Helmholtz-Zentrum Dresden-Rossendorf (HZDR), Dresden, Germany.

Affiliations 2–5 were all condensed under affiliation 2.

The original article has been corrected.
